# A Double‐Blind Randomized Sham‐Controlled Trial of Two Online Cognitive Bias Modification Interventions for Fear of Cancer Recurrence in People With Breast or Ovarian Cancer

**DOI:** 10.1002/pon.70338

**Published:** 2025-11-27

**Authors:** Poorva Pradhan, Louise Sharpe, Hayley Russell, Jemma Todd, Wendy G. Lichtenthal, Courtney Beard, Phyllis Butow

**Affiliations:** ^1^ School of Psychology The University of Sydney Sydney Australia; ^2^ Ovarian Cancer Australia Queen Victoria Women's Centre Melbourne Australia; ^3^ Sylvester Comprehensive Cancer Center University of Miami Health System Miami Florida USA; ^4^ Department of Psychiatry McLean Hospital Harvard Medical School Boston Massachusetts USA

**Keywords:** cancer pain, cognitive bias modification, fear of cancer recurrence, fear of progression, interpretation bias

## Abstract

**Objective:**

In the context of cancer, pain demands interpretation. Our research has found that fear of cancer recurrence (FCR) is associated with the tendency to interpret ambiguous information as health‐related. We aimed to determine whether we could modify these interpretation biases to improve FCR, and pain outcomes.

**Methods:**

We conducted a double‐blind randomized controlled trial comparing two fully automated Cognitive Bias Modification for Interpretation (CBM‐I) programs to a matched sham. We randomized 174 people with breast or ovarian cancer to one of three groups (pain‐related CBM, cancer‐specific CBM or sham). Participants completed four training sessions, and outcomes were assessed before and after intervention and 2 weeks later. We nominated co‐primary outcomes as FCR and fear of progression (FoP) so that measures were suited to those with and without active disease and measured pain outcomes and other secondary psychosocial outcomes.

**Results:**

We analyzed data using mixed‐model linear regression and intention‐to‐treat. Results indicated that both the cancer‐specific and pain‐related training groups showed significant improvements in FCR (*F*_(2,440)_ = 17.19, *p* < 0.0005) and FoP (*F*_(2,440)_ = 15.03, *p* < 0.0005) over time compared to sham. Both versions of CBM were associated with benefits in pain intensity (*F*_(2,440)_ = 6.14, *p* < 0.0005) and pain interference (*F*_(2,440)_ = 5.223, *p* = 0.001) compared to sham. No other secondary outcomes improved.

**Conclusion:**

CBM for interpretation is an efficacious treatment for FCR, FoP and pain outcomes in ovarian and breast cancer. This intervention was delivered wholly online, had high completion rates (80%) and therefore is highly scalable. CBM‐I could be part of a stepped care model to meet the large unmet need for people who are living with and beyond cancer.

## 1 Background

In 2020, there were 19.3 million new cases of cancer globally, and cancer burden is projected to increase to 28.4 million cases by 2040 [1]. Nearly 60% of survivors experience moderate levels of FCR, and 20% experience severe FCR [2]. High levels of FCR are associated with poorer quality of life [3], increased anxiety and depressive symptoms [4], and increased oncology appointments and health care costs [5]. Help with FCR remains the highest psychosocial unmet need among survivors [6]. One consistent finding in the literature is that people with cancer who experience pain and other somatic symptoms experience more FCR [7].

Somatic experiences are part of everyday life. How these experiences are interpreted can impact one's distress, and this is especially true among individuals with a history of cancer. When people experience physical changes in their body, they first interpret whether the sensation constitutes pain (pain‐related interpretation), and then determine whether it is a threat (threat‐related interpretation) [8]. For individuals with a history of cancer, they often immediately worry that pain could be a sign of recurrence or progression (i.e., a cancer‐related interpretation) [9]. These cognitive processes have been termed interpretation biases and are believed play a causal role in the development and maintenance of fear of cancer recurrence or progression (FCR) [9]. People living with or beyond cancer interpret ambiguous information as health‐related more than people who have never had cancer [10]. Cancer survivors with more severe FCR seem to be especially vulnerable to this type of interpretation bias [7, 10, 11]. Indeed, two studies found that the experience of pain was associated with interpretation bias [7, 11]. Importantly, in women with breast cancer, interpretation biases predicted persistent distress in breast cancer patients over 2 years, suggesting their potential causal role in maintaining cancer‐related distress [12]. This raises the question, can these interpretation biases be modified to reduce FCR?

Cognitive bias modification (CBM) is an intervention that modifies interpretation biases by training people to interpret ambiguous situations in a benign manner. In anxiety disorders, CBM is associated with small to moderate effects in reducing anxiety compared to placebo [13]. However, CBM has rarely been used in people with health conditions. Two studies have demonstrated that CBM can reduce negative psychosocial outcomes in chronic pain [14] and can improve pain severity and pain interference compared to a placebo [15]. However, CBM has been used only once in cancer. Lichtenthal and colleagues [16] randomized 120 participants with breast cancer to receive CBM that modified both attention and interpretation of cancer‐related biases. CBM reduced health worries about cancer and modified interpretation (but not attention) biases toward cancer‐related information. As such, they recommended a larger trial of CBM to modify interpretation biases for people with cancer. CBM is brief, can be fully automated and if efficacious, could be highly scalable.

Although there are now efficacious treatments for people with moderate to severe FCR [17], most are intensive face‐to‐face approaches or blended approaches with face‐to‐face sessions [17]. It would be impossible for the psycho‐oncology workforce to meet the needs of all survivors using intensive psychological therapies. However, internet‐delivered options have failed to provide benefit (e.g., CAREST [18]) or been tested only in uncontrolled trials (e.g., iConquerFear [19, 20, 21]). For this reason, we aimed to determine whether CBM was efficacious for FCR compared to a sham.

We compared two fully automated CBM programs with a matched sham condition, one targeting pain‐related interpretation biases (pain‐related CBM) and one targeting cancer‐related interpretation biases (cancer‐specific CBM). Because we were interested in understanding the separate effects of modifying biases associated with pain and modifying biases associated with cancer‐related fear, we decided to compare the efficacy of these two standalone CBM programs with preliminary evidence for efficacy in chronic pain [15] and cancer [16], respectively. We hypothesized that CBM would be efficacious in reducing FCR and FoP. We also expected an improvement with CBM in a range of secondary outcomes compared to sham, including pain severity and pain interference.

## 2 Methods

### 2.1 Study Design

This was a randomized, double blind, sham‐controlled trial. Participants were randomly allocated to one of the three groups, using the randomizer algorithm in QUALTRICS: (1) Pain‐related CBM, (2) Cancer‐specific CBM, or (3) a sham control arm. As such, randomization was concealed throughout the trial from both participants and researchers.

### 2.2 Participants

Participants were recruited from Cancer Consumer registries: Breast Cancer Network Australia (BCNA) and Ovarian Cancer Australia (OCA) or through paid advertisements on social media. We chose breast and ovarian cancer because there is considerable evidence of interpretation biases in these two samples [7, 10, 11] and good breast and ovarian cancer‐specific stimuli had been developed by our prior work. Adult participants were recruited from October 2021 to February 2022 and were eligible if they reported a diagnosis of breast or ovarian cancer, reported at least moderate levels on FCR Inventory—Revised [22] (≥ 13) or Fear of Progression Questionnaire—Short Form (FoPQ [23]) (≥ 34). To take part, participants needed to have access to the internet and be fluent in English. We excluded participants receiving palliative care.

### 2.3 Procedure

Participants who expressed interest were emailed a link to consent and complete baseline questionnaires. All questionnaires and training were hosted on a web‐based platform, QUALTRICS. Neither participants nor researchers were aware of the allocated group until the end of the trial. After completing baseline questionnaires, the first training session was administered. Participants were then sent automated emails for subsequent training sessions (2–4) on days 4, 7 and 14. Training sessions were identical across the four sessions and took 15–20 min. Questionnaires were completed before the first and after the fourth training session and 2 weeks later. The study was approved by University of Sydney's Human Research Ethics Committee (2020/835) and the trial is registered with Australian New Zealand Clinical Trials Registry (ACTRN12621000634875).

### 2.4 Intervention

#### 2.4.1 Cancer‐Specific CBM

A modified version of the Word Sentence Association Paradigm (WSAP) [16, 24] was used to modify interpretation bias using word‐sentence pairings to train participants to make benign interpretations. In the WSAP task, each trial begins with a fixation cross for 500 ms. Participants are presented with a single word or phrase representing the benign or threatening interpretation of a sentence that follows (750 ms). For example, either the phrase “suspicious mass” [cancer‐related] or “thorough” [benign] is presented on the screen. Following the word, an ambiguous sentence is presented, such as “The technician takes additional scans”. The participant is asked whether the two are related (“Yes” or “No”). In CBM, benign interpretations (e.g., thorough) are CORRECT and threat interpretations (e.g., suspicious mass) are INCORRECT, and patients receive feedback on every trial before proceeding to the next trial.

We used 80 sentences, each paired with both a benign or cancer‐related interpretation, for 160 total trials. These scenarios were adapted from Lichtenthal et al. [16] and were developed with input from survivors specific to the type of cancer (i.e., breast or ovarian).

#### 2.4.2 Pain‐Related CBM

For pain‐related CBM, to be pragmatic we used a CBM training that had been found to be efficacious for people with chronic pain [15]. The ambiguous scenarios paradigm was used to train participants to make benign interpretations of potentially pain‐related situations [15]. Participants were presented with 30 ambiguous scenarios, each of which could be resolved in a painful or benign resolution, but none of which were related to cancer. Each scenario was followed by a word fragment. Participants were instructed to imagine themselves in each scenario and to solve the word fragment. To train participants to make benign interpretations, all word fragments represented benign, rather than pain‐related outcomes.

After each scenario, participants were asked a comprehension question and to indicate whether the question was related to the previous scenario (“Yes” or “No”). Participants were then given “correct” feedback when they endorsed a benign (not pain‐related) response. If participants endorsed a painful resolution, they were given “incorrect” feedback. Both CBM tasks were matched for time.

#### 2.4.3 Sham

Participants in the sham condition received the same stimuli as the cancer‐specific CBM group. Participants were given feedback quasi‐randomly on each trial, such that 50% of the trials reinforced a benign association, while 50% reinforced a cancer‐related interpretation. We chose this sham because a similar sham had been used by Lichtenthal and colleagues without any unexpected effects (e.g., worsening or improvement in FCR) and it controlled for exposure to cancer‐related scenarios.

### 2.5 Outcome Measures

All participants received both a measure of FCR and FOPQ.

#### 2.5.1 Fear of Cancer Recurrence Inventory (FCRI) [22]

The FCRI severity subscale is a 9‐item scale used to measure FCR. We used the cut‐off score of 13 to indicate at least moderate FCR [22]. Cronbach's alpha for the current sample was 0.79.

#### 2.5.2 Fear of Progression Questionnaire‐ Short Form (FoPQ) [23]

We also included the 12‐item FoPQ because FCR and FoP might measure related, but different phenomena [25]. A score of 34 or higher has been previously used as a cut‐off for at least moderately high FoP [7, 11, 25]. The Cronbach alpha for this measure was 0.87.

### 2.6 Manipulation Check

#### 2.6.1 Interpretation Bias (IB) Assessment

Cancer‐related interpretation bias was assessed using WSAP [24]. We presented 12 trials of word‐sentence pairings with no feedback to assess endorsement rates for cancer‐related and benign interpretations of ambiguous sentences. The stimuli used in test trials were different to those used in CBM training. The task was otherwise identical to the cancer‐specific training task except no feedback was given after responses. This task was administered at post‐treatment, to reduce practice effects over time [23].

### 2.7 Secondary Outcome Measures

The following secondary measures were administered at baseline, after training and 2 weeks later:

*The Physical Symptoms Inventory* (*PSI*) [26]: This an 18‐item questionnaire where participants indicate whether or not they experience each symptom (during the past 30 days) and if they did, whether they had sought medical attention for it. Symptoms are scored as absent (0), present (1) and/or needed to seek medical attention (2) and summed.

The *Brief Pain inventory* (*BPI* [27]): This brief measure was used to assess the severity of pain (intensity subscale) and its impact on daily functioning (interference subscale).

*Hospital Anxiety and Depression Scale* (*HADS* [28]): Participants' depression and anxiety will be measured using the Hospital Anxiety and Depression scale (HADS). The questionnaire comprises of seven items for anxiety and seven items for depression.

*European Organization for Research and Treatment of Cancer Quality of Life (QoL) Questionnaire* (*EORTC QLQ‐C30* [29]): This will be used to measure QoL. It has 30 items comprising distinct scales, each representing a different aspect of QoL. For the purposes of this study, we analyzed the total QoL score.

### 2.8 Data Analysis

The previous cancer‐related CBM study obtained a treatment‐related effect size of Hedge's *f* = 0.25 [16]. Assuming a similar effect size, we needed 179 participants to have 80% power to detect differences between the groups [30].

Linear mixed model regression (LMMR) analyses were performed to test the efficacy of CBM on the co‐primary outcomes of FCR and FoP, as well as the secondary outcomes. Data were analyzed according to intention‐to‐treat principle and LMMR was used to impute the missing data. In order to determine whether induced interpretation bias mediated the treatment effect, we conducted a mediation analysis with group as the independent variable and FCR and FOP as the dependent variable, and induced interpretation bias as the mediating variable, using the PROCESS macro [31].

We calculated effect sizes based on the differences between baseline and times 2 and 3 between the sham and each individual active treatment. Hence, the formula employed was:

Cohen’sd=(MeanT1CBMI–MeanT2/T3CBMI)–MeanT1sham–MeanT2−T3sham)

### 2.9 Pooled Standard Deviation

We also conducted post‐hoc analyses to determine whether cancer type, stage or status impacted the treatment.

## 3 Results

### 3.1 Participant Characteristics

**FIGURE 1:**
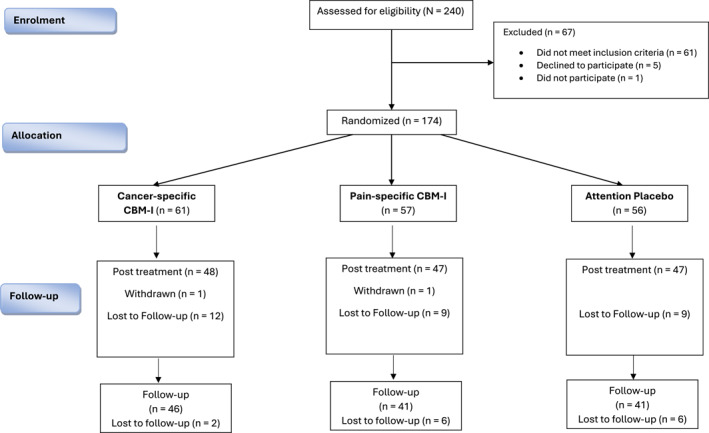
CONSORT diagram of participant flow through the trial.

Two hundred and forty people with breast or ovarian cancer responded to our advertisement and were assessed for eligibility. Sixty‐one did not meet inclusion criteria, leaving 179 eligible participants. Five declined to participate, and 1 consented but did not commence the assessment. Hence, 173 people completed baseline and were randomized: cancer‐specific CBM (*n* = 60), pain‐specific CBM (*n* = 58) or sham (*n* = 56). We had high completion rates at post‐treatment (80%–83%) and follow‐up (71%–77%) (see Figure 1) with no reports of adverse events. Demographic and medical variables are presented in Table 1.

**TABLE 1:** Medical and demographic characteristics of the sample.

Variable	Mean (SD)
Age	58.49 (10.33)
	Frequency (percentage)
Cancer type	
Breast cancer	115 (66.1)
Ovarian cancer	59 (33.9)
Marital status	
Married/De facto	119 (68.4)
Not currently married	55 (31.6)
Children	
None	52 (29.9)
Has children	122 (70.1)
Education level	
Did not complete high school	10 (5.7)
Completed high school	51 (29.3)
Undergraduate degree at university	58 (33.3)
Postgraduate degree at university	55 (31.6)
Employment status	
Currently employed	81 (46.6)
Currently unemployed	93 (53.4)
Stage at diagnosis	
Stage 1	41 (23.6)
Stage 2	46 (26.4)
Stage 3	56 (32.2)
Stage 4	18 (10.3)
Not known	13 (7.5)
Current cancer status	
Currently on treatment	63 (36.2)
No current evidence of disease	95 (54.6)
Current evidence of disease, no current treatment	16 (9.2)
Cancer recurrence	
Yes	41 (23.6)
No	133 (76.4)
Cancer surgery	
Yes	168 (96.6)
No	6 (3.4)
Treatment type (also include participants who received more than one treatment type)	
Radiotherapy	49 (28.2)
Chemotherapy	70 (40.2)
Hormonal therapy	48 (27.6)
Other	43 (24.7)

### 3.2 Manipulation Check

We conducted a 3 (treatment group) x 2 (valence: threat or benign) between subjects ANOVA to examine the differences in post‐intervention interpretation bias for people allocated to each group. Participants allocated to CBM endorsed more benign responses than those allocated to sham (*F*_(2,139)_ = 56.68, *p* < 0.0005). Likewise, both CBM groups made fewer cancer‐specific responses than those allocated to sham, indicating CBM had successfully modified biases.

### 3.3 Main Analyses

**FIGURE 2:**
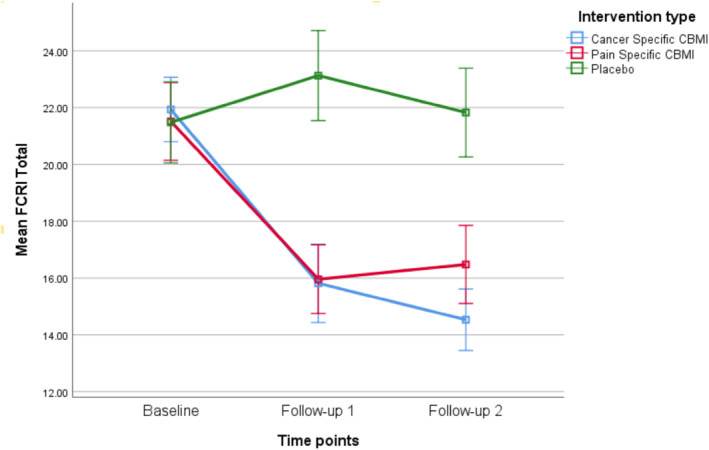
Changes over time in fear of cancer recurrence inventory (FCRI).

**FIGURE 3:**
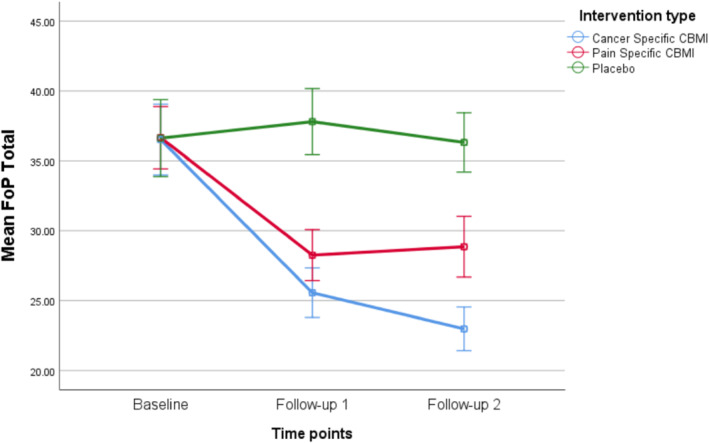
Changes over time in fear of progression questionnaire (FoPQ).

The LMRR for FCR demonstrated a significant interaction effect between time and group, which favored the two CBM groups over sham (*F*_(2,440)_ = 17.19, *p* < 0.0005). Change in FCR over time was significantly different for both of the CBM groups compared to sham at post‐treatment and 2‐week follow‐up (See Figure 2 and Table 2). Likewise, for FoP there was a significant interaction effect between time and group, which favored the two CBM groups compared to sham (*F*_(2,440)_ = 15.03, *p* < 0.0005). The difference in FoP change over time between pre‐ and post‐treatment was significant for both CBM groups compared to sham and at follow‐up (See Figure 3 and Table 2).

**TABLE 2:** Means and standard deviations for each variable and each group, with interaction effect, effect sizes and 95% confidence intervals.

Outcome measures	Cancer specific CBM‐I			Pain specific CBM‐I			Placebo			Cancer specific versus placebo				Pain specific versus. placebo			
	M	SD	*N*	M	SD	*N*	M	SD	*N*	*t*	95% CI (of *t*)	*p*	ES	*t*	95% CI (of *t*)	*p*	ES
FCR																	
Baseline	21.93	4.39	60	21.52	5.20	58	21.48	5.33	56								
Post‐treatment	15.86	4.74	48	16.00	4.12	47	23.11	5.40	47	7.027	5.41, 9.63	< 0.0005	1.43	4.602	2.86, 7.17	< 0.0005	1.48
Follow‐up	14.62	3.68	47	16.68	4.40	42	21.81	4.95	41	7.071	5.51, 9.78	< 0.0005	1.51	4.682	2.99, 7.34	< 0.0005	1.10
FOP																	
Baseline	36.52	9.82	60	36.66	8.48	58	36.63	10.28	56								
Post‐treatment	25.63	6.10	48	28.50	6.19	47	37.67	8.06	47	7.136	9.33, 16.47	< 0.0005	1.68	3.934	3.60, 10.84	< 0.0005	1.27
Follow‐up	23.21	5.33	47	29.21	6.96	42	36.17	6.70	41	7.122	9.29, 16.42	< 0.0005	1.54	3.808	3.37, 10.62	< 0.0005	1.02
Pain intensity																	
Baseline	2.19	2.08	60	2.28	1.88	58	2.44	2.15	56								
Post‐treatment	1.37	1.28	48	0.98	0.97	47	3.26	2.25	47	3.113	0.49, 2.21	0.002	1.03	3.862	0.83, 2.58	< 0.0005	1.31
Follow‐up	1.40	1.27	47	1.13	1.32	42	2.87	2.06	41	2.829	0.37, 2.07	0.005	0.86	3.595	0.71, 2.44	< 0.001	1.00
Pain interference																	
Baseline	3.54	2.43	60	3.52	2.39	58	3.86	2.55	56								
Post‐treatment	1.76	1.45	48	1.14	1.06	47	3.72	2.36	47	2.652	0.35, 2.39	0.009	1.00	3.116	0.60, 2.67	0.002	1.41
Follow‐up	1.55	1.48	47	1.27	1.32	42	3.15	2.12	41	2.478	0.26, 2.30	0.014	0.88	2.925	0.50, 2.58	0.004	1.06
Physical symptoms																	
Baseline	14.48	9.62	60	15.62	10.69	58	14.39	10.33	56								
Post‐treatment	7.95	4.11	48	6.56	3.31	47	9.56	5.47	47	0.142	−3.77, 4.36	0.887	0.33	1.264	−1.48, 6.74	0.208	0.66
Follow‐up	8.03	4.28	47	7.02	4.53	42	7.97	5.39	41	0.016	−3.97, 4.04	0.987	0.01	1.058	−1.88, 6.23	0.292	0.19
Anxiety																	
Baseline	9.78	4.17	60	9.33	3.50	58	10.23	4.84	56								
Post‐treatment	7.68	4.54	48	7.21	3.89	47	8.88	5.21	47	1.146	−0.66, 2.47	0.254	0.24	0.902	−0.87, 2.32	0.369	0.36
Follow‐up	6.87	4.21	47	6.66	3.58	42	7.99	4.28	41	0.852	−0.89, 2.23	0.396	0.26	0.535	−1.16, 2.03	0.535	0.33
Depression																	
Baseline	7.88	4.58	60	6.57	3.94	58	7.63	5.14	56								
Post‐treatment	6.57	4.74	48	5.24	3.65	47	6.95	5.26	47	0.470	−1.37, 2.23	0.639	0.07	−0.165	−1.99, 1.68	0.869	0.37
Follow‐up	5.52	4.48	47	4.78	3.33	42	5.39	3.72	41	0.135	−1.66, 1.90	0.893	0.03	−0.489	−2.26, 1.37	0.626	0.17
Quality of life																	
Baseline	8.80	2.68	60	8.85	2.37	58	8.43	2.30	56								
Post‐treatment	10.25	2.28	48	10.35	1.97	47	9.48	2.42	47	0.464	−0.81, 1.30	0.643	0.32	0.236	−0.95, 1.20	0.814	0.39
Follow‐up	10.31	2.26	47	10.53	2.03	42	10.24	1.82	41	0.573	−0.75, 1.37	0.567	0.03	0.244	−0.95, 1.21	808	0.15

Abbreviations: CI = confidence interval; ES = Effect Size (Cohen's *d)*; FCR = Fear of cancer recurrence; FOP = Fear of progression; M = mean; SD = Standard deviation.

**FIGURE 4:**
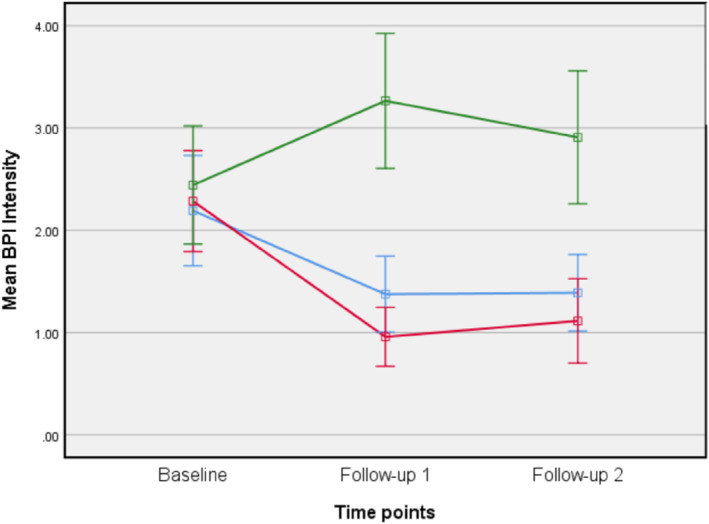
Changes in pain intensity on brief pain inventory (BPI).

**FIGURE 5:**
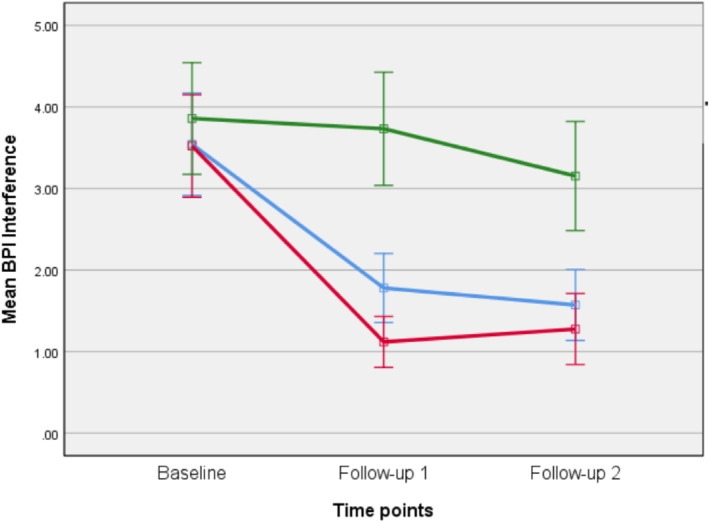
Changes over time in pain interference on the brief pain inventory (BPI).

As for FCR and FOP, the LMMR analyses revealed a significant interaction effect of time by group, favoring both CBM groups for pain intensity (*F*_(2,440)_ = 6.14, *p* < 0.0005). That is, pain intensity was reduced for both CBM groups more than for the sham. Similar results were found for pain interference, where there was also a significant interaction of group by time favoring CBM training over sham (*F*_(2,440)_ = 5.223, *p* = 0.001). Participants in both cancer‐specific CBM and pain‐related CBM had larger reductions in interference compared to sham (See Figures 4 and 5 and Table 2). There were no significant differences between the two CBM groups. None of the remaining secondary outcomes were significantly impacted by CBM training.

### 3.4 Additional Analyses

We conducted mediation analyses to determine whether induced interpretation bias could mediate the relationship between group membership and FCR or FoP at post‐treatment. Mediation analyses were not significant.

To determine whether cancer type (breast or ovarian cancer), cancer status (whether an individual had experienced a recurrence) or cancer stage impacted the effect of CBMI, we conducted a series of mixed‐model linear regression analyses and explored the three‐way interaction between treatment group x time x each cancer‐related variable.

For those who had experienced a recurrence, levels of FCR were higher at baseline (*t* = 6.185, *p* = 0.013). However, participants in the cancer‐related CBMI who had experienced a recurrence improved more over time than in the other two groups (*F* = 15.313, *P* < 0.0005), such that there was no longer a difference between these groups at follow‐up (*t* = 0.630, *p* = 0.427). There were no significant differences in outcome between groups based on women with ovarian versus breast cancer or the stage of cancer at diagnosis.

## 4 Discussion

The present study investigated the efficacy of two CBM training programs, one targeting pain‐related interpretations and one targeting cancer‐specific interpretations, for people with breast or ovarian cancer. Both CBM training programs were associated with predicted differences in how people interpreted ambiguous information, indicating training was successful. CBM demonstrated large improvements in FCR and FoP, as well as pain severity and pain interference, which were maintained 2 weeks later. There were no differences in efficacy between the cancer‐specific and pain‐related CBM. No significant effects of CBM were observed for other outcomes. Nevertheless, participants in this study experienced levels of FCR that were severe [32] and similar to treatment‐seeking samples [16, 18, 19, 20, 21]. Over treatment, FCR reduced by, on average, seven points, which corresponds to a large effect size (Cohen's *d* ≈ 1). The effect is larger than those previously reported in the FCR literature, even with face‐to‐face interventions (Cohen's *d* = 0.38^17^). Moreover, there was no evidence of any difference between treatment efficacy for those with breast versus ovarian cancer or based on the stage of cancer. Interestingly, those who had experienced a recurrence improved more than those who had not, although only with the cancer‐specific CBMI training.

One advantage of CBMI is that it is based on a clear theory that suggests that the way that ambiguous information is interpreted is core to the development of severe levels of FCR [9]. Heathcote and Eccleston's Cancer Threat Interpretation model indicates that there are two types of interpretations that fuel FCR: (1) interpreting whether ambiguous sensations are pain‐related; and (2) whether pain and other ambiguous scenarios are cancer‐related (i.e., threatening). Our two CBMI treatments were equally efficacious on FCR, FoP and pain outcomes. There are two potential interpretations for this finding. First, it may be that both CBM programs are modifying the same mechanism of changing the automatic interpretation of ambiguous information toward a benign rather than threatening interpretation. This explanation would be more compelling had we established that induced interpretation biases mediated the relative efficacy of the CBMI compared to sham. The second interpretation is that both interpretation biases contribute equally to FCR and therefore modifying either will have benefits. While it is also possible that another mechanism, such as exposure, could account for the changes, exposure is unlikely since the sham treatment controlled for the presentation of cancer‐related scenarios. Nevertheless, future research is needed to understand the mechanisms of CBMI.

### 4.1 Clinical Implications

The fact that the more general pain‐related CBM protocol was efficacious means that it is likely that these results would generalize to other cancers, since cancer‐specificity conferred no additional benefit. Theoretically, it has been argued that when ambiguous sensations are interpreted as pain, this leads to cancer‐related interpretations [8] and that by targeting either type of biases similar benefits were produced. Importantly, CBM was fully automated, requiring no therapist time, and an hour over 2 weeks for participants to gain benefit. Even if the benefits were only short‐lasting, there are times (such as anniversaries, waiting for scans, post‐surgically), when a brief intervention could make a clinically meaningful difference to people living with or beyond cancer [33]. Hence, CBM is a potential minimal intervention that could form part of a stepped care model for FCR [34].

This is an important contribution because there are no minimal interventions that have been shown to be efficacious for FCR [35]. Efficacious interventions that require no or minimal therapist time are a good first step for those with moderate to severe FCR, allowing psycho‐oncologists to focus on those with the most complex needs [34]. The demonstrated efficacy of CBM is the first efficacious totally remote intervention with large effects on FCR. Future research should confirm whether CBM‐related benefits are maintained. It is also important to note that the CBM also improved pain severity and pain interference. This is, in and of itself, an important finding. Persistent pain is a problem for nearly 40% of survivors treated with curative intent, and persistent pain is more common during treatment (55%) or in advanced disease (66%) [36]. If CBM‐I reduces pain severity and associated interference, then CBM will have broader applications for people living with and beyond cancer, such as for managing post‐surgical pain.

### 4.2 Study Limitations

The findings from the current study should be interpreted in light of the following limitations. Firstly, the face validity of these CBM has been criticized [37]. Many authors advocate for consumer input and adaptation of stimuli to different populations (see [38]). Only the cancer‐specific CBM had been co‐developed with survivors and personalized to the cancer (i.e., ovarian vs. breast cancer). The other CBM condition was developed in the laboratory [15] specifically for pain and used pragmatically in this trial. It is encouraging that there were no differences in the efficacy of these programs. Secondly, we recruited predominantly women with two cancers and these findings might not generalize to other cancers, particularly those that predominantly affect men, such as prostrate or testicular cancer. Further, since we recruited via advertisement it is unclear how generalizable the sample is to the population from which it was drawn. Future research could explore this question. Finally, as the study duration and follow‐up were brief, it is still not clear whether these effects are long lasting. However, if 1 h of training every 2 weeks could reduce FCR and pain outcomes for the next 2 weeks, this would nevertheless be clinically meaningful and could be particularly helpful at times when FCR is triggered, such as prior to scans and tests. Future research should endeavor to determine the long‐term efficacy of CBM. Finally, we observed larger effect sizes than expected which require replication.

The strength of this study was that CBM was administered entirely online and involved low costs of delivery, rendering the intervention highly scalable. We included people with current disease as well as those who had been treated with curative intent. Good completion rates were observed and CBM was highly efficacious for both primary outcomes and pain outcomes. Hence, CBM could form an early step in a much needed stepped‐care model to meet the growing number of cancer survivors who fear disease recurrence or progression [34].

## 5 Conclusion

In conclusion, as compared to sham, we found two CBM interventions were efficacious in reducing FCR and FoP. CBM was also effective in reducing the pain‐related outcomes of pain intensity and interference. These effects lasted for up to 2 weeks following intervention and required only an hour of participants' time to achieve these benefits. As FCR remains one of the highest unmet psychosocial needs for those living with and beyond cancers, future research should confirm these benefits with other cancer types. Given that CBM was delivered entirely remotely, future research could adapt this online program to a more sophisticated, engaging and smartphone friendly intervention that could be used by people living with and beyond cancer [39].

## Author Contributions

P.P., L.S., H.R., J.T., W.G.L., C.B. and P.B. were involved in the conceptualisation of the study. L.S., W.G.L. and C.B. provided the resources. P.P. was responsible for data curation. L.S. was responsible for software. L.S. and P.P. conducted the formal analysis. L.S. was the recipient of the funding. P.P. conducted the investigation. P.P. and L.S. were responsible for visualisation. P.P., L.S., H.R., J.T., W.G.L., C.B. and P.B. developed the methodology. P.P. oversaw project administration, under the supervision of L.S., P.P. and L.S. wrote the first draft. All authors were involved in the reviewing and editing of the final manuscript.

## Funding

The development of the Cancer‐specific CBM program was funded by National Institutes of Health Center for Translational Science Center (CTSC) grant UL1TR00457 (pilot funding); the T.J. Martell Foundation; and National Cancer Institute grants K07CA172216 (Lichtenthal), P30CA008748 (Vickers), and P30CA240139 (Nimer). This study was funded, in part, by an Australian Research Council Discovery Project to Louise Sharpe (DP21010101827).

## Conflicts of Interest

The authors declare no conflicts of interest.

## Supporting information


Supporting Information S1

## Data Availability

Data available on reasonable request from the corresponding author.
