# Supplementary material for: A Double‐Blind Randomized Sham‐Controlled Trial of Two Online Cognitive Bias Modification Interventions for Fear of Cancer Recurrence in People With Breast or Ovarian Cancer
Source: Psychooncology. 2025 Nov 27;34(12):e70338. doi: 10.1002/pon.70338 (PMC12660550; doi:10.1002/pon.70338)
Supplement: Supplementary file 1 — Supporting Information S1 [file PON-34-e70338-s001.docx]

**Table S1.**

*Example items from the Ambiguous Scenarios Task used in Cognitive Bias Modification for Interpretation*

| **Ambiguous Scenario** | **Word fragment (resolution)** | | **Comprehension question** | | **Comprehension response** | | |  |
| --- | --- | --- | --- | --- | --- | --- | --- | --- |
|  | *Pain group* | *Benign group* | |  | | *Pain group* | *Benign group* | |
| You are driving home on the highway, and without realising you crash into another car. It looks pretty bad. You go over to the car and ﬁnd that the driver is… | inj_r_d  (injured) | a_g_y  (angry) | | Was the driver badly injured? | | Yes | No | |
| You are bush walking. Suddenly, you trip over and fall onto your knees. Your knees feel all wet, and you look down to see… | bl_ _d  (blood) | le_v_s  (leaves) | | Are your knees bleeding? | | Yes | No | |
| You and your friend are preparing dinner together. She cuts the onion and you cut the capsicum. Suddenly, her knife slips, and cuts into your… | fi_g_r  (finger) | c_ps_c_m  (capsicum) | | Did you cut yourself? | | Yes | No | |
| Yesterday, your bicycle was hit by a car. You will not be able to cycle for a while because the car broke your… | l_g  (leg) | b_k_  (bike) | | Is your leg broken after the accident? | | Yes | No | |
| You have been bent over a bucket all morning and are feeling very low on energy. This is because you have been… | vo_it_ng  (vomiting) | cle_n_ _g  (cleaning) | | Were you bent over a bucket because of an illness? | | Yes | No | |
| You ﬁnd that your eyes are sore and swollen. They are so puffy you can barely open them. This is from… | a_l_rg_es  (allergies) | cr_i_g  (crying) | | Did an allergic reaction cause your eyes to swell? | | Yes | No | |
| You’re feeling dizzy and your head is pounding with heat. You’re vaguely aware of background noises around you. This is because you are… | fain_i_g  (fainting) | s_n-b_k_ng  (sun-baking) | | Was your dizziness brought on by the sun? | | No | Yes | |
| You are at a ceremony for your best friend. All of her family and friends are there, and her parents are crying. Your best friend has just gotten… | b_ri_d  (buried) | m_rr_ _d  (married) | | Are you at your best friend’s wedding? | | No | Yes | |
| You are trying to explain something to a friend, but your throat feels increasingly sore and constricted. This is because you… | have a thr_at inf_ct_on  (throat infection) | are  em_t__nal  (emotional) | | Is your throat closed up because of an infection? | | Yes | No | |
| You see your grandma and she tells you she has an appointment with the doctor to look at two spots on her hand. The spots are caused by… | c_nc_r  (cancer) | a_e  (age) | | Has your grandma been diagnosed with cancer? | | Yes | No | |
| You are driving home on the highway, and without realising you crash into another car. It looks pretty bad. You go over to the car and ﬁnd that the driver is… | inj_r_d  (injured) | a_g_y  (angry) | | Was the driver badly injured? | | Yes | No | |
| You catch up with an old friend, but to your surprise, he is completely bald when you see him. This is because he has… | c_nc_r  (cancer) | sh_v_d  (shaved) | | Is your friend bald because he has just shaved? | | No | Yes | |
| You’re in a restaurant. On a table nearby you see a man gasping for air. His body is shaking and his mouth is wide open. He is… | ch_ki_g  (choking) | la_g_ _ng  (laughing) | | Was the man in the restaurant laughing? | | No | Yes | |
| You take your place in the line. On a small table in front of you there are a few magazines. You notice that there are still two people in the queue in front of you. It is annoying that you have to wait this long for the… | do_t_r  (doctor) | ha_r-dr_ss_r  (hair-dresser) | | Were you in the doctor’s waiting room? | | Yes | No | |
| It’s dark outside but you just can’t sleep. You are very aware of the people around you who are keeping you up with their coughing. You are in a… | h_sp_t_l  (hospital) | h_st_l  (hostel) | | Were you kept awake in a hospital bed? | | Yes | No | |
| Beads of sweat are covering your forehead. Your heart is pounding very hard and your breathing is irregular. This is because you are… | p_n_cki_g  (panicking) | j_gg_ng  (jogging) | | Is your heart pounding because you are exercising? | | No | Yes | |
| The woman walks slowly towards you. She seems to be making every effort not to lose her balance. It’s hard for her to walk with… | cr_tch_s  (crutches) | h_gh h_ _ls  (high heels) | | Was the woman struggling to walk with crutches? | | Yes | No | |
| You’re walking on the street. Several meters ahead of you, you see a man slip and fall hard onto the pavement. When you get to him, he is lying in a large puddle of… | b_ _od  (blood) | w_t_r  (water) | | Was the man lying in his own blood? | | Yes | No | |
| You are chatting with an experienced skier. He took part in major international ski competitions. Not so long ago he broke a… | l_g  (leg) | r_c_rd  (record) | | Did the skier injure himself recently? | | Yes | No | |
| The man sitting next to you suddenly jumps right out of his seat, and puts both hands immediately on his chest. He is… | in p_ _n  (pain) | sh_ck_d  (shocked) | | Was the man next to you in pain? | | Yes | No | |
| Your father had cancer. You had to go back every month with him to the hospital for treatment, but that now that no longer happens. This is because he recently… | d_ _d  (died) | r_co_ _red  (recovered) | | Did your father recently pass away? | | Yes | No | |
| You are gasping for air and seem unable to ﬁll your lungs enough. Your chest is moving up and down very quickly, and it’s not getting any easier to breathe. You… | have ast_m_a  (asthma) | are wo_k__g o_t  (working out) | | Is your breathing difficult due to asthma? | | Yes | No | |
| The operation was carefully planned, however, the end result was a disaster. Consequently, the young man had to be taken away by… | par_med_cs  (paramedics) | po_ _ce  (police) | | Was the young man taken by the paramedics? | | Yes | No | |
| You go to visit your grandmother in the nursing home. You ﬁnd her lying in her bed, facing upwards and hardly moving. She is… | dec_a_ _d  (deceased) | sl_ _p_ng  (sleeping) | | Was your grandmother dead when you found her? | | Yes | No | |
| You have a bulge on your foot and you find that wearing shoes is irritating. You go to the doctors and they tell you it is a… | t_mo_r  (tumour) | b_te  (bite) | | Do you have a tumour? | | Yes | No | |
| On the other side of the street is a woman who is struggling with the uneven pavement. She has to use a lot of force to push her… | whe_lcha_r  (wheel chair) | pr_m  (pram) | | Did the woman struggle with her wheelchair? | | Yes | No | |
| Your friend takes a pill every morning at breakfast. The pill is… | med_ _i_e  (medicine) | a v_t_mi_  (vitamin) | | Does your friend need medicine every day? | | Yes | No | |
| Yesterday, your bicycle was hit by a car. You will not be able to cycle for a while because the car broke your… | l_g  (leg) | b_k_  (bike) | | Is your leg broken after the accident? | | Yes | No | |
| You walk across the road because you spot your friend Kim. She has lost a lot of weight and looks thin. This is the result of an… | il_n_ _s  (illness) | d_ _t  (diet) | | Does your friend Kim have a disease? | | Yes | No | |
| You tried to warn Jack, but it was already too late. He fell over and stained the carpet with… | bl_ _d  (blood) | wi_e  (wine) | | Did Jack stain carpet with his blood? | | Yes | No | |
| You’re on a holiday in Brazil and a mosquito stings you. You are worried, and decide to go to the doctor. He tells you that you have caught… | ma_ar_a  (malaria) | n_th_ _ g  (nothing) | | Did you catch malaria from the mosquito? | | Yes | No | |
| You have a shower and then ﬁnish. Once you ﬁnish drying your hair, you hang your towel up and notice it’s covered in red patches. The red patches are… | b_ _od  (blood) | h_ _r-d_e  (hair-dye) | | Did you find blood on your towel? | | Yes | No | |

**Table S2.**

*Items, response options, and coding of response options for the recognition task used to measure interpretation bias*

| **Title** | **Ambiguous Scenario** | **Sentence Resolutions** | **Interpretation Type** |
| --- | --- | --- | --- |
| The iPhone Accident | You are walking across a street in the middle of writing a text to a friend. Because you are not looking where you are going, you bump into a **p_le** [pole] | You bumped into a pole but you’re not hurt and no one saw  You bumped into a pole and now your head is bleeding  You bumped into a pole and a car nearby honked at you  You bumped into a pole and you were embarrassed | Benign-target  Pain-target  Benign-foil  Pain-foil |
| At the Gym | You are doing your weekly work-out at the gym. The guy next to you is really pushing himself, and is panting like crazy. Within minutes of you being there, you hear a loud **so_nd** [sound] | The loud sound is the man celebrating the end of his workout  The loud sound was the man yelling because he had dropped the dumbbell on his toe  The loud sound was another person moving a heavy weight  The loud sound was him shouting because of exhaustion | Benign-target  Pain-target  Benign-foil  Pain-foil |
| The Kitchen | You and your brother are fighting in the kitchen. He says that you do not understand where he is coming from. In his frustration, he slams the fridge  **d_ _r** [door] | (1) Your brother slammed the fridge door and walked away angrily  Your brother slammed the fridge door and jammed your finger  Your brother slammed the fridge door and went silent  Your brother slammed the fridge door and continued to shout | Benign-target  Pain-target  Benign-foil  Pain-foil |
| The Pharmacy | Your hands are dry and itchy. You decide to go to the pharmacist, and amongst the bag of things you have bought is a **cr_ _m** [cream] | The cream you bought is a shaving cream  The cream you bought is medicated, to soothe the pain from eczema  The cream you bought is a daily moisturizer for your face  The cream you bought is a moisturizer to soothe your itchy skin | Benign-target  Pain-target  Benign-foil  Pain-foil |
| Moving House | You are moving house today and are surrounded by boxes of your belongings. Your friend Jack is helping you for the day. You walk into another room, and hear a rather loud **b_ng** [bang] | The bang was Jack knocking something over as he walked past  The bang was Jack dropping a box because he pulled a muscle in his back  The bang was a door slamming because of the wind  The bang was Jack tripping over and falling | Benign-target  Pain-target  Benign-foil  Pain-foil |
| The Road Trip | You and your friend are on a road trip North. You have the music playing loudly, and have plenty of snacks to eat on the journey. Everything is fun until a car in the left lane swerves sharply and causes you to have a **cr_sh** [crash] | The crash wasn’t serious and you and your friend are both okay  The crash was pretty bad and you have whiplash from the sudden braking  The crash wasn’t serious but the car will definitely need fixing  The crash was pretty bad and you’re not sure if the other driver is okay | Benign-target  Pain-target  Benign-foil  Pain-foil |
| The Baguette | You are walking along a busy street eating a baguette for lunch with lots of filling. It’s quite hard to eat, but delicious all the same. You take a new mouth full, and have to stop **ch_w_ng** [chewing] | You stopped chewing because you bumped into someone by accident  You stopped chewing because you bit your tongue whilst eating  You stopped chewing because something caught your eye  You stopped chewing because your jaw was sore | Benign-target  Pain-target  Benign-foil  Pain-foil |
| The Doctors | Your mum has been worried about a lump in her breast for some time now. 2 weeks ago she went to get a mammogram, and you are now in with her talking to the doctor. The doctor politely asks you to leave the **r_ _m** [room] | The doctor asked you to leave due to patient confidentiality  The doctor asked you to leave because he is telling your mum she has cancer  The doctor asked you to leave because you were distracting  The doctor asked you to leave because you may not want to hear what he has to say | Benign-target  Pain-target  Benign-foil  Pain-foil |
| The Thriller Movie | You are watching a thriller movie, and the main character is being hunted down by the murderer. The main character walks slowly around the corner to find herself face to face with the murderer, and she starts to**sc_e_m**[scream] | As the main character turns the corner, the murderer tries to grab her, but she runs away  As the main character turns the corner, she sees the murderer and is stabbed and cries out in pain  As the main character turns the corner she sees the murderer is someone she knows  As the main character turns the corner, she sees the murderer with a knife | Benign-target  Pain-target  Benign-foil  Pain-foil |
| The Water Park | You and your friends decide to go to the water slide park while on your holiday. It is a really fun day, and at the end you are all exhausted. One of your friends forgets to bring a change of clothes. As a result, she is wet and is **sh_v_r_ng** [shivering] | Your friend was shivering, but once you got out of the cold she got warm  Your friend was shivering, and because of her wet clothes she developed a cold  Your friend was shivering, and when you got home her clothes were still wet  Your friend was shivering, and her wet clothes were very uncomfortable | Benign-target  Pain-target  Benign-foil  Pain-foil |

**Table S3.**

*Items and examples of pain and neutral responses for the ambiguous cues task used to measure interpretation bias*

| **Cue Word** | **Example Pain Responses** | **Example Neutral Responses** |
| --- | --- | --- |
| Terminal | Cancer, illness, growth | End, airport, velocity |
| Needle | Blood, injection, shot | Sew, pins, haystack |
| Wheel | Chair, disabled | Bike, drive, spin |
| Plaster | Bandage, fracture, scar | Wall, build, sculpture |
| Growth | Cancer, tumour, pain | Plant, hair, development |
| Wrenching | Cramp, excruciating, vomit | Heart, gut, twisting |
| Block | Pain, nerve, medicine | Mental, brick, apartment |
| Back | Pain, sore, ache | Bend, spine, behind |
| Relief | Pain, medication, symptoms | Anxiety, calm, sigh |
| Nerve | Block, pain, damage | Ending, system, anxious |
| Bed | Ridden, hospital, sores | Room, fatigue, pan |
| Pound | Ache, head, throb | Beat, fight, weight |
| Shot | Injection, flu, needle | Gun, bullet, head |
| Attack | Heart, asthma, disease | Victim, defend, hit |
